# Supplementary material for: HIVprotI: an integrated web based platform for prediction and design of HIV proteins inhibitors
Source: J Cheminform. 2018 Mar 9;10:12. doi: 10.1186/s13321-018-0266-y (PMC5845081; doi:10.1186/s13321-018-0266-y)
Supplement: Supplementary file 2 — Additional file 2. Source code of HIVProtI web server. [file 13321_2018_266_MOESM2_ESM.zip › HIVprotI_Source-code/hivprotI/weka-3-6-6/doc/weka/core/converters/AbstractFileLoader.html]

AbstractFileLoader


---


|  |  |  |
| --- | --- | --- |
| **Overview** \| **Package** \| **Class** \| **Tree** \| **Deprecated** \| **Index** \| **Help** \| **Weka's home** | |  |
| PREV CLASS   **NEXT CLASS** | **FRAMES**    **NO FRAMES**     **All Classes** |  |
| SUMMARY: NESTED \| FIELD \| CONSTR \| METHOD | DETAIL: FIELD \| CONSTR \| METHOD |  |


---


## weka.core.converters Class AbstractFileLoader

```
java.lang.Object
  weka.core.converters.AbstractLoader
      weka.core.converters.AbstractFileLoader
```

**All Implemented Interfaces:**: java.io.Serializable, FileSourcedConverter, Loader, EnvironmentHandler, RevisionHandler

**Direct Known Subclasses:**: ArffLoader, C45Loader, CSVLoader, LibSVMLoader, SerializedInstancesLoader, SVMLightLoader, XRFFLoader

---

``` public abstract class AbstractFileLoader extends AbstractLoader implements FileSourcedConverter, EnvironmentHandler ```

Abstract superclass for all file loaders.

**Version:**
:   $Revision: 7391 $

**Author:**
:   fracpete (fracpete at waikato dot ac dot nz)

**See Also:**: Serialized Form

---

| **Field Summary** | |
| --- | --- |
| `static java.lang.String` | `FILE_EXTENSION_COMPRESSED`             the extension for compressed files |

**Fields inherited from interface weka.core.converters.Loader**

`BATCH, INCREMENTAL, NONE`


**Constructor Summary**

`AbstractFileLoader()`


| **Method Summary** | |
| --- | --- |
| `boolean` | `getUseRelativePath()`             Gets whether relative paths are to be used |
| `void` | `reset()`             Resets the loader ready to read a new data set |
| `java.io.File` | `retrieveFile()`             get the File specified as the source |
| `static void` | `runFileLoader(AbstractFileLoader loader, java.lang.String[] options)`             runs the given loader with the provided options |
| `void` | `setEnvironment(Environment env)`             Set the environment variables to use. |
| `void` | `setFile(java.io.File file)`             sets the source File |
| `void` | `setSource(java.io.File file)`             Resets the Loader object and sets the source of the data set to be the supplied File object. |
| `void` | `setUseRelativePath(boolean rp)`             Set whether to use relative rather than absolute paths |
| `java.lang.String` | `useRelativePathTipText()`             Tip text suitable for displaying int the GUI |

**Methods inherited from class weka.core.converters.AbstractLoader**

`getDataSet, getNextInstance, getStructure, setRetrieval, setSource`

**Methods inherited from class java.lang.Object**

`equals, getClass, hashCode, notify, notifyAll, toString, wait, wait, wait`

**Methods inherited from interface weka.core.converters.FileSourcedConverter**

`getFileDescription, getFileExtension, getFileExtensions`

**Methods inherited from interface weka.core.RevisionHandler**

`getRevision`

**Field Detail**

### FILE\_EXTENSION\_COMPRESSED

```
public static java.lang.String FILE_EXTENSION_COMPRESSED
```

:   the extension for compressed files


**Constructor Detail**

### AbstractFileLoader

```
public AbstractFileLoader()
```


**Method Detail**

### retrieveFile

```
public java.io.File retrieveFile()
```

:   get the File specified as the source

    :   **Specified by:**: `retrieveFile` in interface `FileSourcedConverter`
    :   **Returns:**: the source file

---


### setFile

```
public void setFile(java.io.File file)
             throws java.io.IOException
```

:   sets the source File

    :   **Specified by:**: `setFile` in interface `FileSourcedConverter`
    :   **Parameters:**: `file` - the source file **Throws:**: `java.io.IOException` - if an error occurs

---


### setEnvironment

```
public void setEnvironment(Environment env)
```

:   Set the environment variables to use.

    :   **Specified by:**: `setEnvironment` in interface `EnvironmentHandler`
    :   **Parameters:**: `env` - the environment variables to use

---


### reset

```
public void reset()
           throws java.io.IOException
```

:   Resets the loader ready to read a new data set

    :   **Specified by:**: `reset` in interface `Loader` **Overrides:**: `reset` in class `AbstractLoader`
    :   **Throws:**: `java.io.IOException` - if something goes wrong

---


### setSource

```
public void setSource(java.io.File file)
               throws java.io.IOException
```

:   Resets the Loader object and sets the source of the data set to be
    the supplied File object.

    :   **Specified by:**: `setSource` in interface `Loader` **Overrides:**: `setSource` in class `AbstractLoader`
    :   **Parameters:**: `file` - the source file. **Throws:**: `java.io.IOException` - if an error occurs

---


### useRelativePathTipText

```
public java.lang.String useRelativePathTipText()
```

:   Tip text suitable for displaying int the GUI

    :   **Returns:**: a description of this property as a String

---


### setUseRelativePath

```
public void setUseRelativePath(boolean rp)
```

:   Set whether to use relative rather than absolute paths

    :   **Specified by:**: `setUseRelativePath` in interface `FileSourcedConverter`
    :   **Parameters:**: `rp` - true if relative paths are to be used

---


### getUseRelativePath

```
public boolean getUseRelativePath()
```

:   Gets whether relative paths are to be used

    :   **Specified by:**: `getUseRelativePath` in interface `FileSourcedConverter`
    :   **Returns:**: true if relative paths are to be used

---


### runFileLoader

```
public static void runFileLoader(AbstractFileLoader loader,
                                 java.lang.String[] options)
```

:   runs the given loader with the provided options

    :   **Parameters:**: `loader` - the loader to run: `options` - the commandline options, first argument must be the file to load


---


|  |  |  |
| --- | --- | --- |
| **Overview** \| **Package** \| **Class** \| **Tree** \| **Deprecated** \| **Index** \| **Help** \| **Weka's home** | |  |
| PREV CLASS   **NEXT CLASS** | **FRAMES**    **NO FRAMES**     **All Classes** |  |
| SUMMARY: NESTED \| FIELD \| CONSTR \| METHOD | DETAIL: FIELD \| CONSTR \| METHOD |  |


---
